# Oddioside A, a New Phenolic Glycoside Isolated from the Fruits of *Morus alba* (Mulberry), Protects TNF-α-Induced Human Dermal Fibroblast Damage

**DOI:** 10.3390/antiox11101894

**Published:** 2022-09-24

**Authors:** Kang Sub Kim, Ranhee Kim, So-Ri Son, Ki Sung Kang, Dae Sik Jang, Sullim Lee

**Affiliations:** 1 College of Korean Medicine, Gachon University, Seongnam 13120, Korea; krkdtjq@nate.com (K.S.K.); kkang@gachon.ac.kr (K.S.K.); 2 Department of Life and Nanopharmaceutical Sciences, Graduate School, Kyung Hee University, Seoul 02447, Korea; rhee0423@khu.ac.kr; 3 Department of Biomedical and Pharmaceutical Sciences, Graduate School, Kyung Hee University, Seoul 02447, Korea; allosori@khu.ac.kr; 4 Department of Life Science, College of Bio-Nano Technology, Gachon University, Seongnam 13120, Korea

**Keywords:** human dermal fibroblasts, tumor necrosis factor-α, skin damage, *Morus alba*, phenolic glycoside

## Abstract

In our preliminary study, a hot water extract from the fruits of *Morus alba* (mulberry) inhibited the secretion of metalloproteinase-1 (MMP-1) against tumor necrosis factor-α (TNF-α)-stimulated human dermal fibroblasts (HDFs), and therefore we researched its active compounds. In the present study, a new phenolic glycoside (oddioside A, **1**) and 21 known compounds (**2**−**22**) were isolated from the hot water extract from the fruits of *M. alba* by repeated chromatography. The chemical structure of the new compound **1** was elucidated by its spectroscopic data (1D− and 2D−NMR and HRMS) measurement and by acidic hydrolysis. The presence of sargentodoside E (**2**), eugenyl glucoside (**6**), 2-*O*-β-d-glucopyranosyl-4,6-dihydroxybenzaldehyde **(7**), 7*S*,8*R*-erythro-7,9,9’-trihydroxy-3,3’-dimethoxy-8-*O*-4’-neolignan-4-*O*-β-d-glucopyranoside (**11**), pinoresinol-4-*O*-β-d-glucopyranoside (**12**), taxifolin-7-*O*-β-d-glucopyranoside (**20**), and pinellic acid (**21**) were reported from *M. alba* for the first time in this study. The new compound oddioside A (**1**) suppressed the secretion of MMP-1 and increased collagen in TNF-α-stimulated HDFs. In addition, the phosphorylation of mitogen-activated protein kinases (MAPKs) was inhibited by oddioside A. In conclusion, the extract from fruits of *M. alba* and its constituent oddioside A may be a potential agent to prevent inflammation-related skin aging and other skin disorders.

## 1. Introduction

The skin is an organ that can protect the body from water loss or microbial infection, and is directly affected by external environmental factors such as air pollution and UV irradiations [1]. Continuous exposure to these external factors accelerates skin aging such as deep and shallow wrinkles, sagging, rough and dry skin, and pigmentation. Regarding skin aging, many people spend a lot on cosmetics or therapeutics that prevent or improve aging. This demand for cosmetics promotes research on skin aging [2].

Aging is largely divided into intrinsic aging and extrinsic aging. Intrinsic aging is a process that changes physiologically over time, and extrinsic aging is caused by external environmental factors including tobacco, air pollution, and UV irradiations [3]. In photoaging, symptoms such as wrinkle formation, low elasticity, and rough skin appear due to structural changes in the dermal connective tissue [4].

Long-term exposure of the skin to UV leads to DNA damage, protein denaturation, and reactive oxygen species (ROS) [5]. Excessive ROS generation by UV causes oxidative stress in the skin and promotes the synthesis of matrix metalloproteinases (MMPs). As a result, MMPs degrade the extracellular matrix (ECM) rich in collagen, elastin and proteoglycan to form wrinkles [6,7,8]. Therefore, antioxidants may be candidates for preventing the formation of skin wrinkles by reducing oxidative stress.

UV also inhibits Transforming Growth Factor-beta (TGF-β) signaling through ROS generation and induces proinflammatory mediators such as tumor necrosis factor-alpha (TNF-α), prostaglandin E2 (PGE2), cyclooxygenase-2 (COX-2), interleukin-1 (IL-1) and interleukin-6 (IL-6) receptors. Tumor necrosis factor-alpha (TNF-α), a pro-inflammatory cytokine, plays an important role in cell proliferation, cell death, and inflammatory response [6,9]. TNF-α induces the generation of ROS and stimulate signaling pathways including nuclear factor kappa B (NF-κB), activator protein 1 (AP-1) and mitogen-activated protein kinases (MAPKs) that stimulate the expression of MMPs [10]. Therefore, inflammatory response inhibitors can also be considered substances that prevent the formation of skin wrinkles.

The fruits of *Morus alba* L. (family: Moraceae) is called “Oddi” in Korea and is also known as mulberry in the English-speaking countries [11]. *M. alba* is mostly cultivated in East Asia, particularly Korea and China, and is consumed in a variety of forms, including wine, juice, and jam [12,13]. In East Asia, mulberry is also used in traditional medicine for its various pharmacological effects, including fever reduction, liver and kidney protection, treatment of sore throat, eyesight improvement, and ability to lower blood pressure [11]. The pharmacological value of the fruits of *M*. *alba* is originated in its various important secondary metabolites, such as flavonoids, lignans, coumarins, phenolic compounds, and other compounds [14,15]. Recently, there have been attempts to use fruits of *M*. *alba* as antioxidants [16]. Particularly, moracin isolated from *M*. *alba* reduces oxidative stress in hydrogen peroxide induced stress in skin fibroblast cell line (AH927) [17].

In our preliminary study, a hot water extract from the fruits of *M*. *alba* inhibited the secretion of MMP-1 against tumor necrosis factor-α (TNF-α) stimulated human dermal fibroblasts (HDFs). To investigate the active compounds for inflammation-related skin aging and other skin disorders, chemical constituents were isolated from the hot water extract from the fruits of *M*. *alba* by repeated chromatography. The structures of the isolated compounds were elucidated by interpreting 1D- and 2D-nuclear magnetic resonance (NMR) spectroscopic data analysis and acid hydrolysis. All the isolates were evaluated for their protective effects against TNF-α induced HDFs damage.

## 2. Materials and Methods

### 2.1. General Experimental Procedures

General experimental procedures are provided in the Supplementary Materials.

### 2.2. Plant Materials

The fruits of *Morus alba* L. (Moraceae) were purchased from Mae-Il Oddi farm, Changwon-si, Korea, in June 2017 and authenticated by Professor Dae Sik Jang. A voucher specimen (MOAL-2017) of the raw material has been deposited in the Laboratory of Natural Product Medicine, College of Pharmacy, Kyung Hee University, Seoul, Republic of Korea.

### 2.3. Extraction and Isolation

The fresh fruits of *Morus alba* (5.0 kg) were extracted with 50 L of distilled water at 95~100 °C for 2 h to give a hot water extract (1.0 kg). The extract was chromatographed over Diaion HP-20 (*φ* 9.0 × 43.0 cm) eluting with acetone-H_2_O (from 0:1 to 1:0 *v/v*) to afford 21 fractions (F1~F21).

F5 was subjected to column chromatography (CC) on Sephadex LH-20 (*φ* 4.8 × 48.5 cm) with 40 % acetone to give six subfractions (F5-1~F5-6). F5-6 was separated by silica gel CC (*φ* 2.8 × 26.8 cm) with EtOAc-MeOH-H_2_O (from 9:0.5:0.5 to 7:2.5:0.5 *v/v/v*) to obtain compounds **2** (28.0 mg) and **3** (206.2 mg). F6 was fractionated by Sephadex LH-20 CC (*φ* 4.8 × 43.5 cm) eluting with acetone-H_2_O mixture (3:7, *v/v*) to afford six subfractions (F6-1~F6-6). F6-3 was fractionated further by medium pressure liquid chromatography (MPLC) using a Redi Sep-C18 cartridge (86 g, MeOH-H_2_O = 0:10 to 5:5, *v/v*) to isolate the new compound **1** (5.0 mg). F7 was chromatographed over Sephadex LH-20 (*φ* 5.6 × 52.0 cm) with 40% acetone to give six subfractions (F7-1~F7-6). F7−2 was subjected to silica gel CC (*φ* 3.5 × 29.8 cm, 230−400 mesh) with CH_2_Cl_2_-MeOH-H_2_O solvent system (from 9:0.9:0.1 to 7:2.7:0.3, *v/v/v*) to isolate compound **11** (5.0 mg). F7-3 was separated by silica gel CC (*φ* 3.5 × 29.4 cm, 230−400 mesh) eluting with CH_2_Cl_2_-MeOH-H_2_O (from 9:0.9:0.1 to 7:2.7:0.3, *v/v/v*) to afford compounds **5** (10.0 mg), **17** (2.5 mg), and **22** (1.0 mg). F7-6 was subjected to MPLC using a Redi Sep-C18 cartridge (86 g, MeOH-H_2_O = 1:9 to 5:5 *v/v*) to purify compounds **9** (7.2 mg), **10** (2.7 mg), and **20** (6.2 mg). F8 was fractionated by silica gel CC (*φ* 4.8 × 32.0 cm, 230−400 mesh) with CH_2_Cl_2_-MeOH-H_2_O (from 8:1.8:0.2 to 6:3.6:0.4, *v/v/v*) to afford compounds **4** (6.6 mg), **7** (4.6 mg), and **8** (6.5 mg). Compounds **6** (1.4 mg), **12** (2.8 mg), **14** (17.5 mg), **15** (280.9 mg), and **16** (17.5 mg) were isolated from F11 by silica gel CC (*φ* 4.8 × 37.5 cm, 230−400 mesh) eluting with CH_2_Cl_2_-MeOH-H_2_O (from 6:3.6:0.4 to 0:9:1 *v/v/v*). F12 (3.83 g) was subjected to silica gel CC (*φ* 4.3 × 37.0 cm, 230−400 mesh) using CH_2_Cl_2_-MeOH-H_2_O (from 7:2.7:0.3 to 0:9:1 *v/v/v*) to obtain compound **18** (9.5 mg). F13 was fractionated further by silica gel CC (*φ* 4.0 × 27.8 cm, 230−400 mesh) with CH_2_Cl_2_-MeOH-H_2_O (from 9:0.9:0.1 to 6:3.6:0.4, *v/v/v*) to afford compound **19** (2.7 mg). Finally, compounds **13** (22.7 mg) and **21** (15.3 mg) were purified from F16 by Sephadex LH-20 CC (*φ* 3.4 × 57.7 cm) with 60% acetone.

#### Oddioside A (**1**)

Pale yellow powder; [α]_D_^22^ −95.80 (*c* 0.5, MeOH); UV (Acetonitrile) λ_max_ (log ε) 210 nm (4.15), 234 nm (4.12), 281 nm (3.99), 313 nm (3.93); IR (neat) ν_max_ 3210, 2915, 2331, 1669, 1594 cm^−1^; DART-MS *m/z* 499.14202 [M + Na]^+^ (calcd for C_20_H_28_O_13_Na, 499.14276).

### 2.4. Acidic Hydrolysis of **1** and Sugar Identification

Compound **1** (1.0 mg) was subjected to an acid hydrolysis and the absolute configuration of glucose and rhamnose in **1** was confirmed as d and l, respectively, by the method from Tanaka et al. [18].

### 2.5. Sample Preparations

Compounds (**1**–**22**) were dissolved in dimethyl sulfoxide (DMSO; Sigma-Aldrich, St. Louis, MO, USA) to 10 mM. TNF-α (PeproTech, Rocky Hill, NJ, USA) was dissolved in 1% bovine serum albumin (BSA; Georgiachem, Norcross, GA, USA) solution and stored at −20 °C until use.

### 2.6. Cell Culture

The experiment was conducted using human dermal fibroblasts (HDFs), which were obtained from PromoCell GmbH (Sickingenstr, Heidelberg, Germany). For cell culture, Dulbecco’s modified Eagle medium (DMEM; Corning, Manassas, VA, USA) supplemented with 10% fetal bovine serum (FBS; Atlas, Fort Collins, CO, USA), 1% pen strep (penicillin/streptomycin; Gibco, Grand Island, NY, USA) was used and HDFs were cultured in a humidified incubator maintained at 37 °C with 5% CO_2_.

### 2.7. Cell Viability

HDFs were seeded in 96-well plates at 1 × 10^4^ cells/well and cultured for 24 h. Then, cell medium was replaced with a serum-free condition and incubated overnight. Cells were treated with each concentration (μg/mL or μM) of samples and incubated for 24 h. Then, to measure the cell viability, the supernatant was removed, and 100 μL of 10% EZ-Cytox solution (DoGenBio, Seoul, Korea) in serum-free DMEM was put into each well and incubated for 1 h. Absorbance was measured with a microplate reader (SPARK 10M; Tecan, Männedorf, Switzerland) using a wavelength of 450/600 nm.

### 2.8. Enzyme-Linked Immunosorbent Assay (ELISA)

HDFs were seeded in 48-well plates at 2 × 10^4^ cells/well and incubated for 24 h. Then, cell medium was replaced with a serum-free condition and incubated overnight. After 24 h, non-toxic concentrations of samples were pretreated with HDFs for 1 h, followed by treatment with 20 ng/mL TNF-α for 15 min. Absorbance was measured with a microplate reader (SPARK 10M) using a wavelength of 450/600 nm.

### 2.9. ROS Assay

HDFs were seeded in 96-well plates at 1 × 10^4^ cells/well and incubated for 24 h. Then, cell medium was replaced with a serum-free condition and incubated overnight. After 24 h, non-toxic concentrations of sample were pretreated with HDFs for 1 h, followed by treatment with 20 ng/mL TNF-α and 10 μM dichlorofluorescein diacetate (DCFDA; Sigma-Aldrich) for 15 min. After 15 min of incubation, washing with Dulbecco’s phosphate-buffered salines (DPBS; Welgene, Gyeongsangbuk-do, Korea) and fluorescence was measured with a microplate reader (SPARK 10M) using a wavelength of excitation and emission 485/535 nm.

### 2.10. Quantitative Real-Time Polymerase Chain Reaction (qRT-PCR)

HDFs were seeded in 48-well plates at 3 × 10^5^ cells/well and incubated for 24 h. Then, cell medium was replaced with a serum-free condition and incubated overnight. After 24 h, non-toxic concentrations of sample were pretreated with HDFs for 1 h, followed by treatment with 20 ng/mL TNF-α for 24 h. The RNeasy Mini Kit (Qiagen, Germantown, MD, USA) was used to extract total RNA, and the RevertAid First Strand cDNA Synthesis Kit (Thermo Fisher Scientific, Eugene, OR 97402, USA) was used to reverse transcribing the RNA into cDNA. cDNA was amplified using AccuPower^®^ 2X GreenStar™ qPCR Master Mix (Bioneer, Daejeon, Korea), the primers in Table 1, and the QuantStudio 3 real-time PCR system (Applied Biosystems, Foster City, CA, USA). qPCR amplification conditions were as follows: 50 °C 2 min; 95 °C for 10 min; followed by 40 cycles of 95 °C for 15 s; 60 °C for 1 min; and 95 °C for 15 s; 60 °C for 1 min; 95 °C for 15 s. The primer sequences were shown Table 1.

### 2.11. Western Blotting

HDFs were seeded in 6-well plates at a density of 3 × 10^5^ cells/well and cultured for 24 h. Then, cell medium was replaced with a serum-free condition and incubated overnight. After 24 h, 5, 10 and 50 μM compounds were pretreated with HDFs for 1 h, followed by 20 ng/mL TNF-α for 15 min or 6 h. Protein expression levels of p-ERK, ERK, p-JNK, JNK, p-p38, p-38, p-NF-κB, NF-κB and GAPDH by treatment with TNF-α for 15 min were determined, and COX-2 and GAPDH were detected by treatment for 6 h. Before being lysed with 1× radioimmunoprecipitation assay (RIPA) buffer (Tech & Innovation, Gangwon, Korea), cells were washed with DPBS. After centrifugation at 13,000 rpm, 4 °C, the supernatant was used to detect the protein concentration through the BCA Protein Assay Kit (Thermo scientific, Waltham, MA, USA). The same amount of protein was separated using sodium dodecyl sulfate–polyacrylamide gel electrophoresis (SDS-PAGE) and transferred to a polyvinylidene difluoride (PVDF) membrane. Membrane was blocked with 5% skim milk in TBS-Tween20 (TBS-T; Thermo Fisher Scientific). The primary and secondary antibodies were diluted in 1% BSA solution and each reacted at 4 °C overnight and at room temperature for 2 h. To measure the protein bands, SuperSignal^®^ West Femto Maximum Sensitivity Chemiluminescent Substrate (Thermo Fisher Scientific) and Fusion Solo Chemiluminescence System (PEQLAB Biotechnologie GmbH, Erlangen, Germany) were used and quantified using Image J program (TotalLab, Newcastle, UK).

### 2.12. Statistical Analyses

Experimental results were analyzed with GraphPad Prism version 8.0.0 (GraphPad Software Inc., La Jolla, CA, USA) statistical program and expressed as mean ± standard deviation of the mean (SEM). The statistical significance of each group was evaluated by Tukey’s test at the level of *p* < 0.05 after analysis by one-way ANOVA.

## 3. Results

### 3.1. Effects of the Hot Water Extract on the Viability and MMP-1 Secretion of HDFs

**Figure 1:**
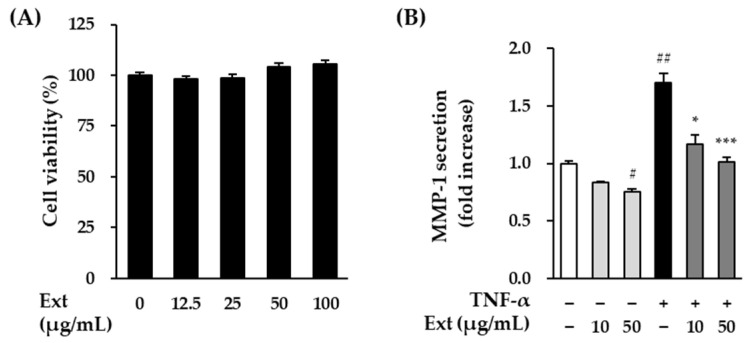
The effects of the hot water extract from the fruits of M. alba on HDFs cell viability (**A**) and MMP-1 secrection (**B**). (**A**) The cells were seeded on 96-well plate with the density of 1 × 10^4^ cells/well and incubated for 24 h. Next, the cells were treated with indicated concentrations of sample for 24 h. The Ez-Cytox kit was used to assess the viability of the cells. (**B**) The cells were seeded on 48-well plate with the density of 2 × 10^4^ cells/well and starved with non-serum media for 24 h. Next, before being exposed to 20 ng/mL TNF-α for 24 h, the cells were first given the relevant sample concentrations to use for 1 h. The MMP-1 secretion in supernatants were determined using ELISA kit. The data were described as mean ± SEM. ^#^ *p* < 0.05 and ^##^ *p* < 0.01 non-treatment group versus TNF-α treatment group. * *p* < 0.05 and *** *p* < 0.001 extract treatment group versus TNF-α treatment group.

Prior to analyze the anti-skin aging effect of the hot water extract from the fruits of M. alba, we investigated the non-toxic concentration of extract on HDFs. As shown in Figure 1A, the extract was not toxic at 12.5~100 μg/mL. Subsequently, we investigated whether the extract could prevent skin aging in TNF-a-stimulated HDFs. The level of MMP-1 secretion to the extract was evaluated at concentrations of 50 μg/mL or less. In Figure 1B, TNF-α treatment group significantly increased the secretion of MMP-1 by 1.70 ± 0.07-folds (*p* < 0.01) compared to the non-treatment group. The extract from the fruits of M. alba exhibited the inhibitory effects on the increase in MMP-1 induced by TNF-α.

### 3.2. Stucture Elucidation of Compound ***1*** and Identification of the Isolates

**Figure 2:**
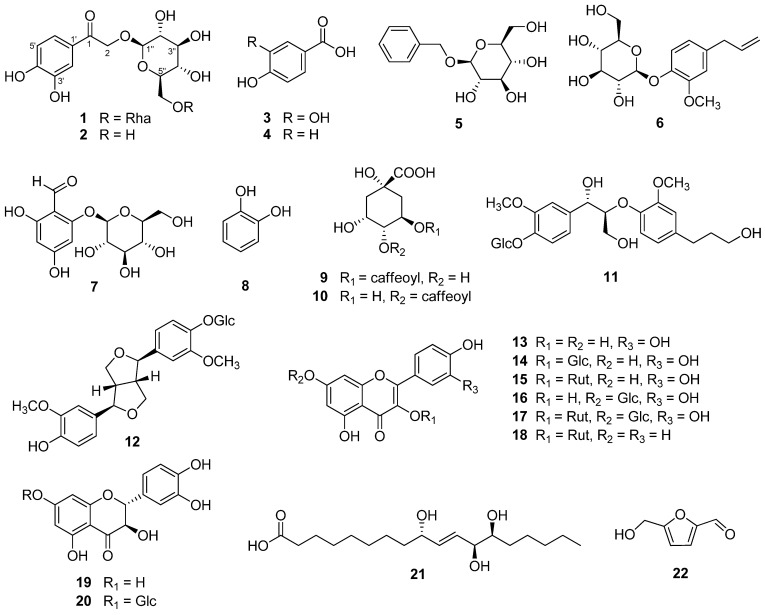
Structures of compounds **1**–**22** isolated from the fruits of *M. alba*.

In the present study, a new phenolic glycoside (**1**) and 21 known compounds (**2**–**22**) were isolated from the fruits of *M*. *alba* (Figure 2).

**Figure 3:**
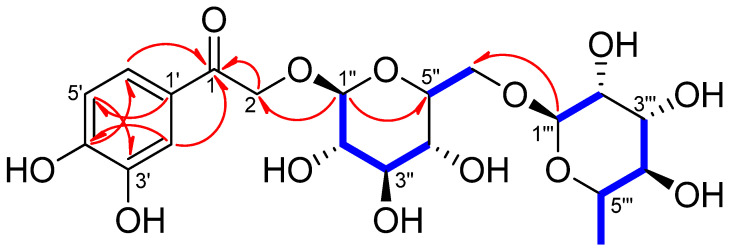
Key ^1^H−^1^H COSY (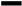) and HMBC (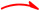) correlations of compound **1**.

Compound **1** exhibited a [M + Na]^+^ ion peak at *m/z* 499.14202 [M + Na]^+^ (calculated for C_20_H_28_O_13_Na, 499.14276) in its HR-DART-MS (Figure S1). The ^1^H-NMR spectrum of **1** exhibited the presence of a 1,3,4-trisubstituted aromatic moiety [*δ*_H_ 7.42 (1H, d, *J* = 2.0 Hz, H-2′), 6.84(1H, d, *J* = 8.5 Hz, H-5′), and 7.44 (1H, dd, *J* = 8.0, 2.0 Hz, H-6′)], an oxymethylene [*δ*_H_ 4.90 (1H, overlapped, H-2b), 5.14 (1H, d, *J* = 17.5 Hz, H-2a)], and two anomeric protons [*δ*_H_ 4.38 (1H, d, *J* = 7.5 Hz, H-Glc-1); and 4.75 (1H, d, *J* = 1.5 Hz, H-Rha-1)] (Table 2 and Figure S2). The ^13^C-NMR spectrum of **1** showed 20 carbon signals including one carbonyl signal (*δ*_C_ 196.8, C-1; Figures S3 and S4). The C-1 was correlated with the aromatic ring protons [*δ*_H_ 7.42 (1H, d, *J* = 2.0 Hz, H-2′) and 7.44 (1H, dd, *J* = 8.0, 2.0 Hz, H-6′)] in the ^1^H-^13^C heteronuclear multiple bond correlation (HMBC) spectrum of **1** (Figure S6). Based on the ^1^H- and ^13^C-NMR, ^1^H-^1^H correlation spectroscopy (COSY), and HMBC (Table 2 and Figure 3 and Figure S5), it was inferred that compound **1** is a phenylethanone glycoside bearing a *β*-glucose and a *α*-rhamnose. The positions of the sugars were determined at C-2 and Glc-6′′ by analysis of the HMBC correlations; H-Glc-1′′ [δ_H_ 4.38 with C-2 (*δ*_C_ 72.2) and H-Rha-1′′′(*δ*_H_ 4.75) with C-Glc-6′′ (*δ*_C_ 68.3). The absolute configurations of the glucose and rhamnose in **1** were identified as d and l forms, respectively, based on the HPLC analysis and acid hydrolysis. The ^1^H- and ^13^C-NMR spectroscopic data of **1** were very similar to those of sargentodoside E (**2**) isolated from *Sargentodoxa cuneata* (Lardizabalaceae) [19] except for the presence of an additional *α*-l-rhamnose unit at Glc-6′′ in **1**. Therefore, the chemical structure of compound **1** was determined to be 1-(3,4-dihydroxyphenyl)-2-hydroxyethanone-2-*O*-rutinoside and named oddioside A.

The structures of the known compounds were identified as sargentodoside E (**2**) [19], protocatechuic acid (**3**) [20], *p*-hydroxybenzoic acid (**4**) [21], benzyl-*O*-*β*-d-glucopyranoside (**5**) [22], eugenyl glucoside (**6**) [23], 2-*O*-*β*-d-glucopyranosyl-4,6-dihydroxybenzaldehyde (**7**) [24], catechol (**8**) [25], chlorogenic acid (**9**) [26], cryptochlorogenic acid (**10**) [27], (7*S*,8*R*)-*erythro*-7,9,9’-trihydroxy-3,3’-dimethoxy-8-*O*-4’-neolignan-4-*O*-*β*-d-glucopyranoside (**11**) [27], pinoresinol-4-*O*-*β*-d-glucopyranoside (**12**) [28], quercetin (**13**) [29], isoquercitrin (**14**) [30], rutin (**15**) [31], quercimeritrin (quercetin-7-*O*-*β*-d-glucopyranoside) (**16**) [32], morkotin A (**17**), nicotiflorin (**18**) [33], taxifolin (**19**) [34], taxifolin-7-*O*-*β*-d-glucopyranoside (**20**) [35], pinellic acid (**21**) [18] and 5-hydroxymethylfurfural (**22**) [36] by comparison of their NMR spectroscopic data with those previously reported data.

### 3.3. Effects of Compounds Isolated from the Fruits of M. alba on the Viability of HDFs

**Figure 4:**
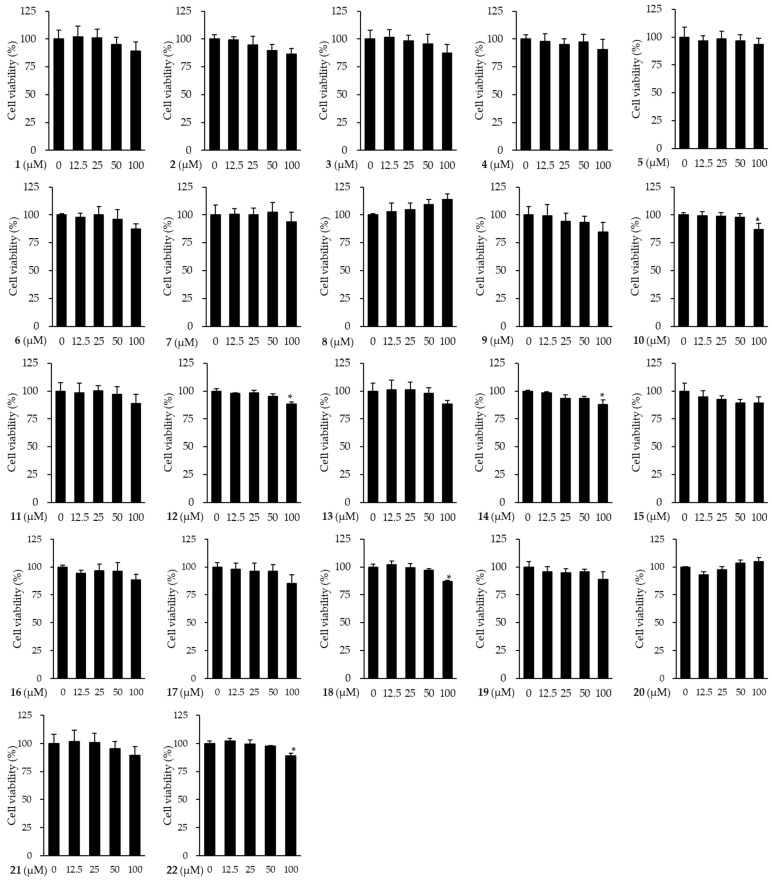
The effects of the compounds (**1**–**22**) isolated from the fruits of M. alba on cell viability of HDFs. The cells were seeded on 96-well plate with the density of 1 × 10^4^ cells/well and incubated for 24 h. Next, the cells were treated with indicated concentration of sample for 24 h. The Ez-Cytox kit was used to assess the viability of the cells. The data were depicted as mean ± SEM. * *p* < 0.05 non-treatment group versus compound treatment groups.

Prior to analyze the anti-skin aging effect of compounds (**1**–**22**) isolated from *M. alba*, we investigated the non-toxic concentrations of compounds to be used in subsequent experiments on HDFs. As shown in Figure 4, among the isolates, five compounds were shown significant reduction in cell viability at 100 μM (**10**: 87.08 ± 5.16%, *p* < 0.01; **12**: 88.42 ± 1.72%, *p* < 0.001; **14**: 87.91 ± 3.97%, *p* < 0.01; **18**: 86.89 ± 1.13%, *p* < 0.001; **22**:88.98 ± 2.11%, *p* < 0.01). Subsequently, experiments for MMP-1 inhibitory effect were performed at 50 μM or less to compare all compounds under the same conditions.

### 3.4. Effects of Compounds Isolated from the Fruits of M. alba on MMP-1 Secretion in TNF-α Induced HDFs

Next, we investigated whether the isolates (**1**–**22**) can prevent skin aging in TNF-α-stimulated HDFs. Base on the cell viability results, the level of MMP-1 secretion to the compounds was screened at concentrations of 50 μM or less.

**Figure 5:**
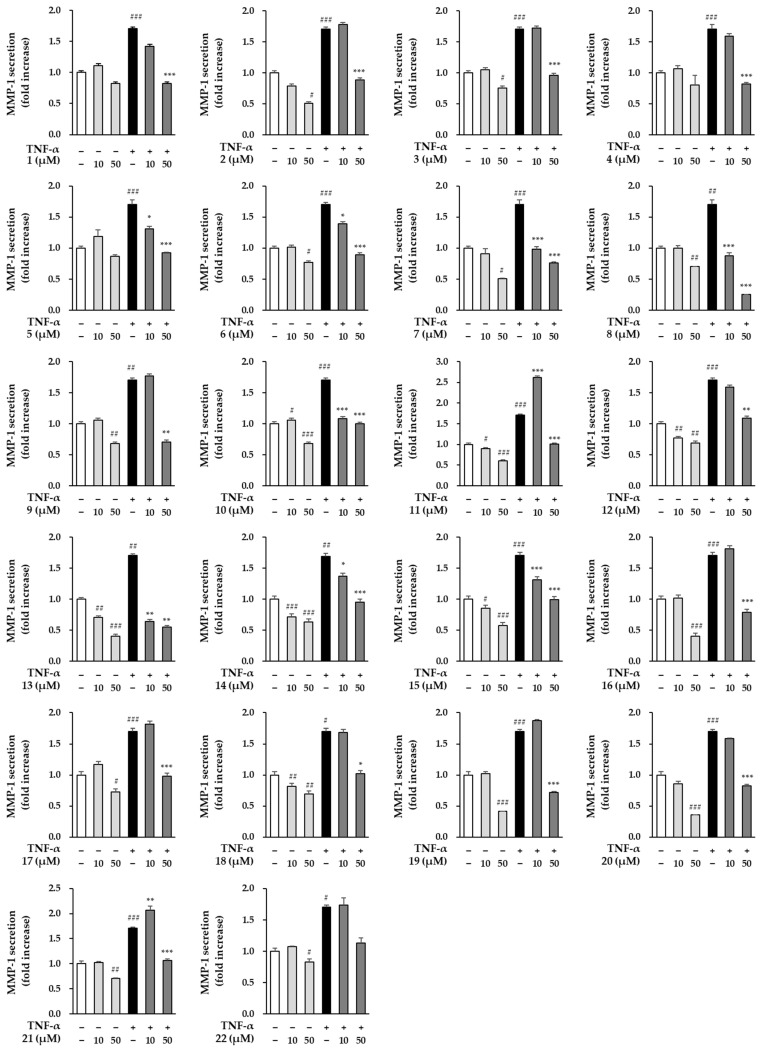
The effects of compounds (**1**–**22**) isolated from the fruits of M. alba on MMP-1 secretion of HDFs. The cells were seeded on 48-well plate with the density of 2 × 10^4^ cells/well and starved with non-serum media for 24 h. Next, before being exposed to 20 ng/mL TNF-α for 24 h, the cells were first given the relevant sample concentrations to use for 1 h. The MMP-1 secretion in supernatants were determined using ELISA kit. The data were described as mean ± SEM. ^#^ *p* < 0.05, ^##^ *p* < 0.01 and ^###^ *p* < 0.001 non-treatment group versus TNF-α treatment group. * *p* < 0.05, ** *p* < 0.01 and *** *p* < 0.001 compound treatment group versus TNF-α treatment group.

In Figure 5, TNF-α treatment group significantly increased the secretion of MMP-1 by 1.70 ± 0.07-folds (*p* < 0.01) compared to the non-treatment group. All compounds isolated from the fruits of M. alba showed significant inhibitory effect on TNF-α-induced MMP-1 increase at a concentration of 50 μM. Table 3 shows the EC_50_ (the concentration of compounds that produce 50% biological effect) against the inhibition of MMP-1 secretion by compounds **1**–**22**. There are nine compounds with EC_50_ values of 20 μM or less, and compounds **1**, **5**, **6**, **7**, **8**, **10**, **13**, **14**, and **15** are applicable. Especially, the new phenolic compound, oddioside A (**1**) showed a significant inhibition of MMP-1 at 50 µM by 0.82 ± 0.02-folds (*p* < 0.001), and the EC_50_ value was calculated as 18.0 μM. Although several compounds isolated from the fruits of M. alba have MMP-1 inhibitory effects on MMP-1, further experiments were conducted focusing on the anti-aging activity of the new compound (**1**).

### 3.5. Effects of Compounds ***1*** on COLIA1 Protein Expression and ROS Production in TNF-α Induced HDFs

**Figure 6:**
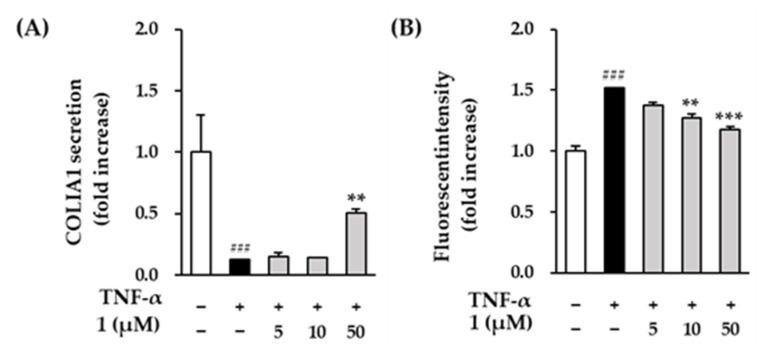
The effects of compound **1** on COLIA1 secretion (**A**) and ROS production (**B**) in TNF-α treated HDFs. (**A**) The cells were seeded on 48-well plate with the density of 2 × 10^4^ cells/well and starved with non-serum media for 24 h. Next, before being exposed to 20 ng/mL TNF-α for 24 h, the cells were first given the relevant sample concentrations to use for 1 h. The COLIA1 secretion in supernatants were determined using ELISA kit. (**B**) The cells were seeded on 96-well plate with the density of 1 × 10^4^ cells/well and starved with non-serum media for 24 h. Next, the cells were treated with indicated concentration of samples for 1 h before exposure to 20 ng/mL TNF-α and 10 µM DCFDA. MMP-1 and COLIA1 mRNA was assessed using qRT-PCR analysis. The data were described as mean ± SEM. ^###^ *p* < 0.001 non-treatment group versus TNF-α treatment group. ** *p* < 0.01 and *** *p* < 0.001 sample treatment group versus TNF-α treatment group.

MMP-1 is a collagenase that plays an important role in the degradation of collagen, and MMP-1 inhibitors can improve collagen reduction caused by external factors such as UV. Therefore, we measured the levels of the procollagen COLIA1 to investigate the effect of compound **1** on collagen reduction. In Figure 6A, TNF-α treatment significantly decreased the protein secretion of COLIA1 compared to the control group (0.12 ± 0.00-folds, *p* < 0.001). The secretion of COLIA1 was significantly increased at the 50 µM of compound **1** (0.50 ± 0.03-folds, *p* < 0.01) compared to the TNF-α-treatment group.

Excessive UV and TNF-α cause oxidative stress such as ROS overproduction, promotes synthesis of MMPs and leads to collagen degradation. Because compound **1** reversed the reduction of collagen by TNF-α, we investigated the inhibitory effect of ROS by compound **1**. In Figure 6B, the TNF-α-treatment group significantly increased ROS production by 1.52 ± 0.00-folds (*p* < 0.001) compared to the control group. Compound **1** showed a significant reduction in a concentration-dependent manner (10 µM: 1.27 ± 0.03-folds, *p* < 0.01; 50 µM: 1.17 ± 0.03-folds, *p* < 0.001).

### 3.6. Effects of Compound ***1*** on Phosphorylation of MAPKs in TNF-α Induced HDFs

**Figure 7:**
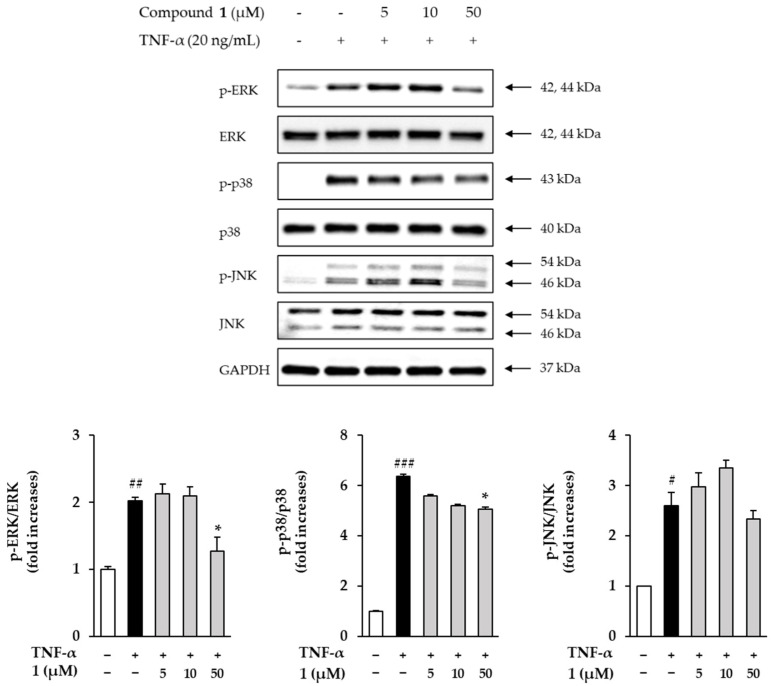
The effects of compound **1** on phosphorylation of MAPKs in TNF-α induced HDFs. The cells were seeded on 6-well plate with the density of 3 × 10^5^ cells/well and starved with non-serum media for 24 h. Next, before being exposed to 20 ng/mL TNF-α for 15 min, the cells were treated with 5, 10 and 50 M of compound 1 for 1 h. Expression of p-ERK, ERK, p–p38, p38, p-JNK, JNK and GAPDH were determined using Western blotting. The data were described as mean ± SEM. # *p* < 0.05, ^##^ *p* < 0.01 and ^###^ *p* < 0.001 non-treatment group versus TNF-α treatment group. * *p* < 0.05 sample treatment group versus TNF-α treatment group.

Next, the effects of compound **1** on phosphorylation of MAPKs in TNF-α-induced HDFs were investigated. The expression of MAPKs was determined using Western blotting. In Figure 7, the ERK phosphorylation of ERK was increased by 2.02 ± 0.04-folds (*p* < 0.01) in the TNF-α treatment group compared to the control group and was inhibited by 1.27 ± 0.21-folds at 50 µM in the compound **1** treatment group. The phosphorylation of p38 was increased by 6.38 ± 0.06-folds (*p* < 0.001) in the TNF-α treatment group compared to the control group and was inhibited by 5.05 ± 0.10-folds at 50 µM in the compound **1** treatment group. The phosphorylation of JNK was increased by 2.60 ± 0.26-folds in the TNF-α treatment group compared to the control group but was not inhibited at 50 µM in the compound **1** treatment group. The TNF-α treatment group induced phosphorylation of ERK, JNK and p38 compared to the control group, and the compound **1** treatment group showed a tendency to inhibit phosphorylation of ERK and p38 at a concentration of 50 µM.

### 3.7. Effects of Compound ***1*** on NF-κB and COX-2 in TNF-α Treated HDFs

**Figure 8:**
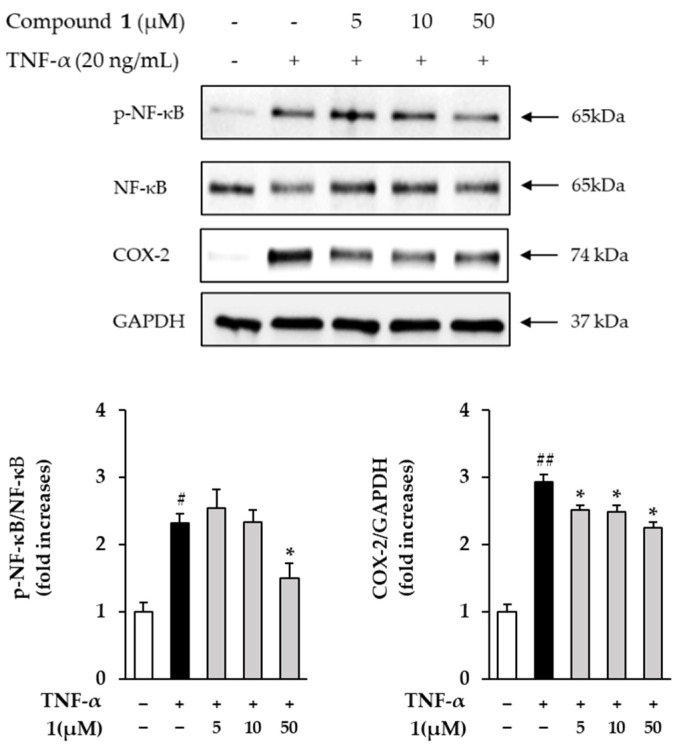
The effects of compound **1** on NF-κB and COX-2 in TNF-α-treated HDFs. The cells were seeded on 6-well plate with the density of 3 × 10^5^ cells/well and starved with non-serum media for 24 h. Next, before being exposed to 20 ng/mL TNF-α for 15 min and 6 h, the cells were treated with 5, 10 and 50 M of compound 1 for 1 h. Expression of NF-κB, COX-2 and GAPDH were determined using Western blotting. The data were described as mean ± SEM. ^#^ *p* < 0.05 and ^##^ *p* < 0.01 and non-treatment group versus TNF-α treatment group. * *p* < 0.05 sample treatment group versus TNF-α treatment group.

Next, the effects of compound **1** on NF-κB and COX-2 in TNF-α-treated HDFs were investigated. The expression of NF-κB and COX-2 was measured using Western blotting. In Figure 8, the phosphorylation of NF-κB was increased by 2.32 ± 0.114-folds in the TNF-α treatment group compared to the control group and was inhibited by 1.50 ± 0.22-folds at 50 µM in the compound **1** treated roup. The expression of COX-2 was increased by 2.94 ± 0.10-folds (*p* < 0.05) in the TNF-α treatment group compared to the control group and was inhibited by 2.25 ± 0.08-folds (*p* < 0.05) at 50 µM in the compound **1** treatment group.

## 4. Discussion

The skin is an organ that is greatly affected by external environmental factors such as ultraviolet rays, stress, and chemicals, and aging can be visually observed [8]. The skin is composed of the epidermis and the dermis, which communicate in various ways to establish, maintain, or restore tissue homeostasis. Dermal tensile strength and elasticity are defined as the properties of the extracellular matrix (ECM) with type I and type III collagen fibrils, microfibrils and elastic fibers [37].

Aging includes intrinsic aging and extrinsic aging, and UV, one of the various causes of extrinsic aging, causes photoaging of the skin. Photoaging induces ROS and pro-inflammatory cytokines such as TNF-α in skin cells and promotes the production of collagen-degrading enzymes, MMPs [38]. MMPs degrade ECM proteins including type 1 collagen, elastin, and fibronectin [39]. ROS and TNF-α activate the phosphorylation of ERK, p38 and JNK and activate the phosphorylation of two subunits of activator protein-1 (AP-1), c-Fos and c-Jun. It also activates and induces phosphorylation and translocation of nuclear factor kappa-light-chain-enhancer of activated B cells (NF-κB) [40,41].

The role of natural products as therapeutic agents has been recognized since ancient times and contributed greatly to various therapeutics, including anti-inflammatory, anti-cancer and anti-diabetic [42]. Among the natural compounds, flavonoids and phenolic compounds have been reported in many reports to contribute to antioxidant and anti-inflammatory activities [43,44,45,46,47]. The fruits of *M. alba* contain a variety of secondary metabolites such as flavonoids, lignans, coumarins, phenolic compounds and other compounds [16]. Among these, the constituents such as catechol, rutin and quercetin have been reported to have antioxidant effects [48,49]. Therefore, the ameliorating effect of the fruits of *M. alba* on inflammation-related skin aging and other skin diseases was investigated in this study. The hot water extract showed an inhibitory effect on the secretion of MMP-1 on TNF-α-stimulated HDFs. Therefore, it was expected that the hot water extract from the fruits of *M. alba* would contain active constituents. A new phenolic glycoside (oddioside A, **1**) and 21 known compounds (**2**−**22**) were isolated from the hot water extract from the fruits of *M. alba* by repeated chromatography in the present work. To the best of our knowledge, the presence of sargentodoside E (**2**), eugenyl glucoside (**6**), 2-*O*-*β*-d-glucopyranosyl-4,6-dihydroxybenzaldehyde (**7**), (7*S*,8*R*)-*erythro*-7,9,9’-trihydroxy-3,3’-dimethoxy-8-*O*-4’-neolignan-4-*O*-*β*-d-glucopyranoside (**11**), pinoresinol-4-*O*-*β*-d-glucopyranoside (**12**), taxifolin-7-*O*-*β*-d-glucopyranoside (**20**) and pinellic acid (**21**) in *M*. *alba* is reported for the first time in this study.

The fruit of *M. alba* is known to be rich in flavonoids, phenolic acid, tannins and stilbenes, and has been reported to have antioxidant, anti-obesity and anti-cancer effects [11,50,51]. The effects of compounds (**1**–**22**) isolated from the fruits of *M. alba* on the secretion of MMP-1 were investigated using TNF-α and HDFs. All compounds significantly reduced MMP-1 secretion at 50 μM without cytotoxicity. Among these, oddioside A (**1**) and compounds **5**, **6**, **7**, **8**, **10**, **13**, **14** and **15** showed strong MMP-1 inhibitory activity. Several compounds isolated in the present study have already been reported for various effects such as anti-inflammatory (**9**, **10**, **13**, **14**, **15**, **16**), antioxidant (**3**, **4**, **5**, **8**, **9**, **13**, **14**, **15**, **19**), and anti-aging (**3**, **9**, **13**) in various cell lines [52,53,54,55,56,57,58,59,60,61,62,63,64,65,66]. Thus, further experiments were focused on the new compound **1** to investigate its potential as a bioactive compound.

Type 1 collagen is mainly distributed in the skin, and type 3 collagen is distributed throughout the body, and the expression level decreases as aging progresses [67,68]. Procollagen, a precursor of collagen, plays an important role in maintaining the elasticity and support structure of the skin by promoting the synthesis of collagen protein [40]. For this reason, the expression level of collagen can be predicted through the investigation of procollagen type 1. Therefore, the effect of compound **1** treatment on the amount of type 1 collagen that affects skin wrinkles was investigated. In Figure 6A, it was measured that the secretion of COLIA1 decreased due to TNF-α was restored with 50 μM of compound **1**.

ROS is one of the well-known causes of inflammation in intrinsic and extrinsic aging, and the inflammatory response accelerates skin aging [69,70]. In addition, ROS promotes the synthesis of MMPs, leading to the breakdown of collagen. One of the MMPs enzymes, MMP-1, is consequently It causes anger, which leads to the formation of wrinkles, loss of elasticity, and sagging [71]. Therefore, the use of antioxidants, one of the many effects of natural products and medicinal plants in the cosmetic industry, is one of the main approaches in skin care product development [72,73]. In this paper, ROS generation was investigated to verify the antioxidant effect of compound **1**, and as a result, compound **1** showed an inhibitory effect on TNF-α-induced ROS generation (Figure 6B).

Many previous studies have revealed that the level of MMP plays an important role in regulating skin damage by NF-κB, AP-1 and MAPKs signaling pathways [74,75,76]. MAPKs signaling pathways include ERK, JNK and p38, and phosphorylation of MAPKs influences phosphorylation of NF-κB and AP-1. In this paper, phosphorylation of ERK, JNK and p38 was induced in the TNF-α-treatment group compared to the control group, and ERK and p38 phosphorylation was inhibited in the 50 µM of compound **1** treatment group (Figure 7). NF-κB is a protein complex that plays an important role in the immune response and can be activated by UV, ROS and pro-inflammatory cytokines [10,77]. In addition, activated NF-κB induces collagen degradation by promoting MMP expression [78]. Next, COX-2 converts arachidonic acid into prostaglandins such as prostaglandin E_2_ (PGE_2_) and is a pro-inflammatory mediator that induces skin aging and inflammatory skin diseases [79,80]. In the present work, it was investigated whether compound **1** had an anti-inflammatory effect. The results showed that phosphorylation of NF-κB and COX-2 was induced in the TNF-α treatment group compared to the control group, and phosphorylation of NF-κB and COX-2 decreased in the group treated with 50 µM compound **1** (Figure 8).

## 5. Conclusions

In this study, the effect of TNF-α induced photoaging in HDFs was investigated using 22 isolated compounds from the fruits of *M. alba*, which is known to have antioxidant effects. Among them, oddioside A (**1**), a new phenolic glycoside, inhibited the secretion of MMP-1 and increased the secretion of type 1 procollagen (COLIA1) compared to the TNF-α treatment group. Compound **1** showed a significant decrease when ROS produced during skin aging and skin damage was induced from HDFs to TNF-α. In addition, compound **1** showed a tendency to inhibit in TNF-α induced HDFs phosphorylation of ERK and p38. Moreover, compound **1** inhibited the phosphorylation of NF-κB and COX-2, which are important for immune and inflammatory responses. Although further experiments are needed to understand the mechanism on skin aging, the fruit of *M. alba* and compound **1** could be used as a natural material to prevent photoaging of the skin.

## Figures and Tables

**Table 1:** Primer sequences.

Gene	Sense Primer Sequence (5’-3’)	Antisense Primer Sequence (5’-3’)
MMP-1	ATTCTACTGATATCGGGGCTTT	ATGTCCTTGGGGTATCCGTGTA
COLIA1	CTCGAGGTGGACACCACCCT	CAGCTGGATGGCCACATCGG
β-actin	AGGAGAAGCTGTGCTACGTC	GGATGTCCACGTCACACTTC

**Table 2:** ^1^H and ^13^C NMR spectroscopic data of compound **1** (*δ* in ppm, methanol-*d*_4_, 500 and 125 MHz).

Position *^a^*	1	
	*δ*_H_ (multi *J* in Hz)	δ_C_
1		196.8
2a 2b	5.14, d, 1H (17.5) 4.90, 1H, overlapped	72.2
1′		128.1
2′	7.42, dd, 1H (2.0)	116.3
3′		146.9
4′		153.1
5′	6.84, d, 1H (8.5)	115.8
6′	7.44, dd, 1H (8.0, 2.0)	123.2
Glc-1″	4.38, d, 1H (7.5)	104.6
Glc-2″	3.32, m	75.2
Glc-3″	3.44, m	77.8
Glc-4″	3.30, overlapped	71.7
Glc-5″	3.28, overlapped	77.3
Glc-6″	3.98, dd, 1H (11.5, 2.0) 3.63, dd, 1H (11.5, 6.5)	68.3
Rha-1‴	4.75, d, 1H (1.5)	102.6
Rha-2‴	3.68, m	70.0
Rha-3‴	3.86, dd, 1H (3.5, 1.5)	72.4
Rha-4‴	3.64, m	72.6
Rha-5‴	3.38, m	74.2
Rha-6‴	1.23, d, 3H (6.0)	18.3

*^a^* Determined by HSQC, COSY, and HMBC results.

**Table 3:** The effect of compounds (**1**–**22**) isolated from the fruits of M. alba on inhibition of MMP-1 secretion.

TNF-α	Comp.	Conc. (µM)	MMP-1 Secretion (Fold Increase)	EC_50_ (µM)	Comp.	Conc. (µM)	MMP-1 Secretion (Fold Increase)	EC_50_ (µM)
− +	− −	− −	1.00 ± 0.03 1.70 ± 0.07					
+	**1**	10 50	1.42 ± 0.12 0.82 ± 0.02	18.0	**12**	10 50	1.59 ± 0.04 1.09 ± 0.03	29.0
+	**2**	10 50	1.78 ± 0.00 0.89 ± 0.00	25.6	**13**	10 50	0.64 ± 0.04 0.55 ± 0.01	8.8
+	**3**	10 50	1.72 ± 0.00 0.96 ± 0.05	26.7	**14**	10 50	1.37 ± 0.07 0.95 ± 0.03	19.1
+	**4**	10 50	1.59 ± 0.04 0.82 ± 0.03	21.1	**15**	10 50	1.31 ± 0.04 0.99 ± 0.02	19.1
+	**5**	10 50	1.31 ± 0.04 0.93 ± 0.00	17.7	**16**	10 50	1.81 ± 0.03 0.79 ± 0.01	23.9
+	**6**	10 50	1.39 ± 0.00 0.89 ± 0.03	18.7	**17**	10 50	1.81 ± 0.03 0.98 ± 0.01	28.3
+	**7**	10 50	0.98 ± 0.04 0.76 ± 0.02	11.3	**18**	10 50	1.68 ± 0.08 1.02 ± 0.03	28.0
+	**8**	10 50	0.87 ± 0.04 0.25 ± 0.00	6.5	**19**	10 50	1.87 ± 0.01 0.71 ± 0.02	23.2
+	**9**	10 50	1.77 ± 0.10 0.70 ± 0.04	21.8	**20**	10 50	1.59 ± 0.00 0.83 ± 0.02	21.2
+	**10**	10 50	1.09 ± 0.03 1.00 ± 0.02	15.6	**21**	10 50	2.07 ± 0.07 1.07 ± 0.02	31.6
+	**11**	10 50	2.62 ± 0.04 1.01 ± 0.03	29.8	**22**	10 50	1.73 ± 0.11 1.13 ± 0.08	33.2

EC_50_: the concentration of compound that produces 50% inhibitory effect.

## Data Availability

Data is contained within the article and supplementary material.

## Supplementary Materials

The following supporting information can be downloaded at: https://www.mdpi.com/2076-3921/11/10/1894/s1, General experimental procedures; Acidic Hydrolysis of **1** and Sugar Identification; Figure S1. HR-DART-MS spectrum of compound **1**; Figure S2. ^1^H-NMR spectrum of compound **1** (500 MHz, methanol-*d*_4_); Figure S3. ^13^C-NMR spectrum of compound **1** (125 MHz, methanol-*d*_4_); Figure S4. ^1^H ^13^C HSQC spectrum of compound **1**; Figure S5. ^1^H ^1^H COSY spectrum of compound **1**; Figure S6. ^1^H ^13^C HMBC spectrum of compound **1**.

## References

[1] Zhang S., Duan E. (2018). Fighting against skin aging: The way from bench to bedside. Cell Transplant..

[2] Cao C., Xiao Z., Wu Y., Ge C. (2020). Diet and skin aging—From the perspective of food nutrition. Nutrients.

[3] Huertas A.C.M., Schmelzer C.E., Hoehenwarter W., Heyroth F., Heinz A. (2016). Molecular-level insights into aging processes of skin elastin. Biochimie.

[4] Kim M., Park H.J. (2016). Molecular mechanisms of skin aging and rejuvenation. Mol. Mech. Aging Process Rejuvenation.

[5] Jeon S., Choi M. (2018). Anti-inflammatory and anti-aging effects of hydroxytyrosol on human dermal fibroblasts (HDFs). Biomed. Dermatol..

[6] Chen S., He Z., Xu J. (2020). Application of adipose-derived stem cells in photoaging: Basic science and literature review. Stem Cell Res. Ther..

[7] Ding Y., Jiratchayamaethasakul C., Lee S.-H. (2020). Protocatechuic Aldehyde Attenuates UVA-induced Photoaging in Human Dermal Fibroblast Cells by Suppressing MAPKs/AP-1 and NF-κB Signaling Pathways. Int. J. Mol. Sci..

[8] Tu Y., Quan T. (2016). Oxidative stress and human skin connective tissue aging. Cosmetics.

[9] Parrado C., Mercado-Saenz S., Perez-Davo A., Gilaberte Y., Gonzalez S., Juarranz A. (2019). Environmental stressors on skin aging. Mechanistic insights. Front. Pharmacol..

[10] Wang L., Lee W., Oh J.Y., Cui Y.R., Ryu B., Jeon Y.-J. (2018). Protective effect of sulfated polysaccharides from celluclast-assisted extract of *Hizikia fusiforme* against ultraviolet B-Induced skin damage by regulating NF-κB, AP-1, and MAPKs signaling pathways in vitro in human dermal fibroblasts. Mar. Drugs.

[11] Yuan Q., Zhao L. (2017). The Mulberry (*Morus alba* L.) Fruit—A Review of Characteristic Components and Health Benefits. J. Agric. Food Chem..

[12] Zhang H., Ma Z.F., Luo X., Li X. (2018). Effects of mulberry fruit (*Morus alba* L.) consumption on health outcomes: A mini-review. Antioxidants.

[13] Huang L., Zhou Y., Meng L., Wu D., He Y. (2017). Comparison of different CCD detectors and chemometrics for predicting total anthocyanin content and antioxidant activity of mulberry fruit using visible and near infrared hyperspectral imaging technique. Food Chem..

[14] Ercisli S., Orhan E. (2007). Chemical composition of white (*Morus alba*), red (*Morus rubra*) and black (*Morus nigra*) mulberry fruits. Food Chem..

[15] Lee S.R., Park J.Y., Yu J.S., Lee S.O., Ryu J.-Y., Choi S.-Z., Kang K.S., Yamabe N., Kim K.H. (2016). Odisolane, a novel oxolane derivative, and antiangiogenic constituents from the fruits of mulberry (*Morus alba* L.). J. Agric. Food Chem..

[16] Memon A.A., Memon N., Luthria D.L., Bhanger M.I., Pitafi A.A. (2010). Phenolic acids profiling and antioxidant potential of mulberry (*Morus laevigata* W., *Morus nigra* L., *Morus alba* L.) leaves and fruits grown in Pakistan. Pol. J. Food Nutr. Sci..

[17] Khyade V.B., Pawar S.S., Khyade R.V. (2018). Oxidative Stress Reducing capabilities of Moracin, the Novel Compound from the Fruits of Mulberry, *Morus alba* (L.) in Hydrogen Peroxide Induced Stress in Skin Fibroblast Cell Line Culture (AH927). Int. J. Sci. Stud..

[18] Shin J.-S., Hong Y., Lee H.-H., Ryu B., Cho Y.-W., Kim N.-J., Jang D.S., Lee K.-T. (2015). Fulgidic acid isolated from the rhizomes of Cyperus rotundus suppresses LPS-induced iNOS, COX-2, TNF-α, and IL-6 expression by AP-1 inactivation in RAW264. 7 macrophages. Biol. Pharm. Bull..

[19] Zeng X., Wang H., Gong Z., Huang J., Pei W., Wang X., Zhang J., Tang X. (2015). Antimicrobial and cytotoxic phenolics and phenolic glycosides from *Sargentodoxa cuneata*. Fitoterapia.

[20] Gutzeit D., Wray V., Winterhalter P., Jerz G. (2007). Preparative isolation and purification of flavonoids and protocatechuic acid from sea buckthorn juice concentrate (*Hippophaë rhamnoides* L. ssp. rhamnoides) by high-speed counter-current chromatography. Chromatographia.

[21] Wang M., Kikuzaki H., Zhu N., Sang S., Nakatani N., Ho C.-T. (2000). Isolation and structural elucidation of two new glycosides from sage (*Salvia officinalis* L.). J. Agric. Food Chem..

[22] De Marino S., Festa C., Zollo F., Iorizzi M. (2009). Phenolic glycosides from *Cucumis melo* var. inodorus seeds. Phytochem. Lett..

[23] Takeda Y., Ooiso Y., Masuda T., Honda G., Otsuka H., Sezik E., Yesilada E. (1998). Iridoid and eugenol glycosides from *Nepeta cadmea*. Phytochemistry.

[24] Li J., Yuan C., Pan L., Benatrehina P.A., Chai H., Keller W.J., Naman C.B., Kinghorn A.D. (2017). Bioassay-guided isolation of antioxidant and cytoprotective constituents from a maqui berry (*Aristotelia chilensis*) dietary supplement ingredient as markers for qualitative and quantitative analysis. J. Agric. Food Chem..

[25] Li L., Seeram N.P. (2010). Maple syrup phytochemicals include lignans, coumarins, a stilbene, and other previously unreported antioxidant phenolic compounds. J. Agric. Food Chem..

[26] Wong S.K., Lim Y.Y., Ling S.K., Chan E.W.C. (2014). Caffeoylquinic acids in leaves of selected Apocynaceae species: Their isolation and content. Pharmacogn. Res..

[27] Hattori M. (2002). NII-electronic library service. Chem. Pharm. Bull..

[28] Kim D.K., Lim J.P., Kim J.W., Park H.W., Eun J.S. (2005). Antitumor and antiinflammatory constituents *Fromceltis sinensis*. Arch. Pharmacal Res..

[29] Lin S., Zhu Q., Wen L., Yang B., Jiang G., Gao H., Chen F., Jiang Y. (2014). Production of quercetin, kaempferol and their glycosidic derivatives from the aqueous-organic extracted residue of litchi pericarp with *Aspergillus awamori*. Food Chem..

[30] Yun J., Lee H., Ko H.J., Woo E.-R., Lee D.G. (2015). Fungicidal effect of isoquercitrin via inducing membrane disturbance. Biochim. Biophys. Acta (BBA) Biomembr..

[31] Liu Q., Zhang Y.-J., Yang C.-R., Xu M. (2009). Phenolic antioxidants from green tea produced from *Camellia crassicolumna* Var. multiplex. J. Agric. Food Chem..

[32] Li M., Han X., Yu B. (2003). Facile synthesis of flavonoid 7-O-glycosides. J. Org. Chem..

[33] Kazuma K., Noda N., Suzuki M. (2003). Malonylated flavonol glycosides from the petals of *Clitoria ternatea*. Phytochemistry.

[34] Baderschneider B., Winterhalter P. (2001). Isolation and characterization of novel benzoates, cinnamates, flavonoids, and lignans from Riesling wine and screening for antioxidant activity. J. Agric. Food Chem..

[35] Pan H., Lundgren L.N. (1996). Phenolics from inner bark of *Pinus sylvestris*. Phytochemistry.

[36] Walia M., Sharma U., Agnihotri V.K., Singh B. (2014). Silica-supported boric acid assisted conversion of mono-and poly-saccharides to 5-hydroxymethylfurfural in ionic liquid. RSC Adv..

[37] Breitkreutz D., Koxholt I., Thiemann K., Nischt R. (2013). Skin basement membrane: The foundation of epidermal integrity—BM functions and diverse roles of bridging molecules nidogen and perlecan. BioMed Res. Int..

[38] Marcos-Garcés V., Molina Aguilar P., Bea Serrano C., García Bustos V., Benavent Seguí J., Ferrández Izquierdo A., Ruiz-Saurí A. (2014). Age-related dermal collagen changes during development, maturation and ageing–a morphometric and comparative study. J. Anat..

[39] Binic I., Lazarevic V., Ljubenovic M., Mojsa J., Sokolovic D. (2013). Skin ageing: Natural weapons and strategies. Evid. Based Complementary Altern. Med..

[40] Hwang E., Gao W., Xiao Y.k., Ngo H.T., Yi T.H. (2019). Helianthus annuus L. flower prevents UVB-induced photodamage in human dermal fibroblasts by regulating the MAPK/AP-1, NFAT, and Nrf2 signaling pathways. J. Cell. Biochem..

[41] Phung H.M., Lee S., Hong S., Lee S., Jung K., Kang K.S. (2021). Protective Effect of Polymethoxyflavones Isolated from *Kaempferia parviflora* against TNF-α-Induced Human Dermal Fibroblast Damage. Antioxidants.

[42] Yuan G., Wahlqvist M.L., He G., Yang M., Li D. (2006). Natural products and anti-inflammatory activity. Asia Pac. J. Clin. Nutr..

[43] Hendra R., Ahmad S., Oskoueian E., Sukari A., Shukor M.Y. (2011). Antioxidant, anti-inflammatory and cytotoxicity of *Phaleria macrocarpa* (Boerl.) Scheff fruit. BMC Complementary Altern. Med..

[44] Zhang L., Ravipati A.S., Koyyalamudi S.R., Jeong S.C., Reddy N., Smith P.T., Bartlett J., Shanmugam K., Münch G., Wu M.J. (2011). Antioxidant and anti-inflammatory activities of selected medicinal plants containing phenolic and flavonoid compounds. J. Agric. Food Chem..

[45] Gülcin I. (2012). Antioxidant activity of food constituents: An overview. Arch. Toxicol..

[46] Gião M.S., González-Sanjosé M.L., Rivero-Pérez M.D., Pereira C.I., Pintado M.E., Malcata F.X. (2007). Infusions of Portuguese medicinal plants: Dependence of final antioxidant capacity and phenol content on extraction features. J. Sci. Food Agric..

[47] Ravipati A.S., Zhang L., Koyyalamudi S.R., Jeong S.C., Reddy N., Bartlett J., Smith P.T., Shanmugam K., Münch G., Wu M.J. (2012). Antioxidant and anti-inflammatory activities of selected Chinese medicinal plants and their relation with antioxidant content. BMC Complementary Altern. Med..

[48] Iacopini P., Baldi M., Storchi P., Sebastiani L. (2008). Catechin, epicatechin, quercetin, rutin and resveratrol in red grape: Content, in vitro antioxidant activity and interactions. J. Food Compos. Anal..

[49] Dudonne S., Poupard P., Coutiere P., Woillez M., Richard T., Merillon J.-M., Vitrac X. (2011). Phenolic composition and antioxidant properties of poplar bud (*Populus nigra*) extract: Individual antioxidant contribution of phenolics and transcriptional effect on skin aging. J. Agric. Food Chem..

[50] Manganaris G.A., Goulas V., Vicente A.R., Terry L.A. (2014). Berry antioxidants: Small fruits providing large benefits. J. Sci. Food Agric..

[51] Chen C., Mohamad Razali U.H., Saikim F.H., Mahyudin A., Mohd Noor N.Q.I. (2021). *Morus alba* L. plant: Bioactive compounds and potential as a functional food ingredient. Foods.

[52] Kakkar S., Bais S. (2014). A review on protocatechuic acid and its pharmacological potential. Int. Sch. Res. Not..

[53] Farhoosh R., Johnny S., Asnaashari M., Molaahmadibahraseman N., Sharif A. (2016). Structure–antioxidant activity relationships of *o*-hydroxyl, *o*-methoxy, and alkyl ester derivatives of *p*-hydroxybenzoic acid. Food Chem..

[54] Luyen B.T.T., Tai B.H., Thao N.P., Yang S.Y., Cuong N.M., Kwon Y.I., Jang H.D., Kim Y.H. (2014). A new phenylpropanoid and an alkylglycoside from *Piper retrofractum* leaves with their antioxidant and α-glucosidase inhibitory activity. Bioorganic Med. Chem. Lett..

[55] Barclay L.R.C., Edwards C., Vinqvist M.R. (1999). Media effects on antioxidant activities of phenols and catechols. J. Am. Chem. Soc..

[56] Girsang E., Ginting C.N., Lister I.N.E., yashfa Gunawan K., Widowati W. (2021). Anti-inflammatory and antiaging properties of chlorogenic acid on UV-induced fibroblast cell. PeerJ.

[57] Sato Y., Itagaki S., Kurokawa T., Ogura J., Kobayashi M., Hirano T., Sugawara M., Iseki K. (2011). In vitro and in vivo antioxidant properties of chlorogenic acid and caffeic acid. Int. J. Pharm..

[58] Ma X., Okyere S.K., Hu L., Wen J., Ren Z., Deng J., Hu Y. (2022). Anti-Inflammatory Activity and Mechanism of Cryptochlorogenic Acid from *Ageratina adenophora*. Nutrients.

[59] Lesjak M., Beara I., Simin N., Pintać D., Majkić T., Bekvalac K., Orčić D., Mimica-Dukić N. (2018). Antioxidant and anti-inflammatory activities of quercetin and its derivatives. J. Funct. Foods.

[60] Reyes-Farias M., Carrasco-Pozo C. (2019). The anti-cancer effect of quercetin: Molecular implications in cancer metabolism. Int. J. Mol. Sci..

[61] Chondrogianni N., Kapeta S., Chinou I., Vassilatou K., Papassideri I., Gonos E.S. (2010). Anti-ageing and rejuvenating effects of quercetin. Exp. Gerontol..

[62] Rogerio A., Kanashiro A., Fontanari C., Da Silva E., Lucisano-Valim Y., Soares E., Faccioli L. (2007). Anti-inflammatory activity of quercetin and isoquercitrin in experimental murine allergic asthma. Inflamm. Res..

[63] Valentová K., Vrba J., Bancířová M., Ulrichová J., Křen V. (2014). Isoquercitrin: Pharmacology, toxicology, and metabolism. Food Chem. Toxicol..

[64] Ganeshpurkar A., Saluja A.K. (2017). The pharmacological potential of rutin. Saudi Pharm. J..

[65] Ahn J.-S., Kwon Y.-S., Kim C.-M. (1999). Anti-inflammatory constituents of Polygonum bistorta. Korean J. Pharmacogn..

[66] Topal F., Nar M., Gocer H., Kalin P., Kocyigit U.M., Gülçin İ., Alwasel S.H. (2016). Antioxidant activity of taxifolin: An activity–structure relationship. J. Enzym. Inhib. Med. Chem..

[67] Liu T., Li N., Yan Y.q., Liu Y., Xiong K., Liu Y., Xia Q.m., Zhang H., Liu Z.d. (2020). Recent advances in the anti-aging effects of phytoestrogens on collagen, water content, and oxidative stress. Phytother. Res..

[68] Lovell C., Smolenski K., Duance V., Light N., Young S., Dyson M. (1987). Type I and III collagen content and fibre distribution in normal human skin during ageing. Br. J. Dermatol..

[69] Fuller B. (2019). Role of PGE-2 and other inflammatory mediators in skin aging and their inhibition by topical natural anti-inflammatories. Cosmetics.

[70] Franceschi C., Campisi J. (2014). Chronic inflammation (inflammaging) and its potential contribution to age-associated diseases. J. Gerontol. Ser. A Biomed. Sci. Med. Sci..

[71] Freitas-Rodriguez S., Folgueras A.R., Lopez-Otin C. (2017). The role of matrix metalloproteinases in aging: Tissue remodeling and beyond. Biochim. Biophys. Acta (BBA) Mol. Cell Res..

[72] Škrovánková S., Mišurcová L., Machů L. (2012). Antioxidant activity and protecting health effects of common medicinal plants. Adv. Food Nutr. Res..

[73] Ganceviciene R., Liakou A.I., Theodoridis A., Makrantonaki E., Zouboulis C.C. (2012). Skin anti-aging strategies. Derm. Endocrinol..

[74] Hwang E., Lin P., Ngo H.T., Gao W., Wang Y.-S., Yu H.-S., Yi T.-H. (2018). Icariin and icaritin recover UVB-induced photoaging by stimulating Nrf2/ARE and reducing AP-1 and NF-κB signaling pathways: A comparative study on UVB-irradiated human keratinocytes. Photochem. Photobiol. Sci..

[75] Pittayapruek P., Meephansan J., Prapapan O., Komine M., Ohtsuki M. (2016). Role of matrix metalloproteinases in photoaging and photocarcinogenesis. Int. J. Mol. Sci..

[76] Rabe J.H., Mamelak A.J., McElgunn P.J., Morison W.L., Sauder D.N. (2006). Photoaging: Mechanisms and repair. J. Am. Acad. Dermatol..

[77] Makarov S.S. (2000). NF-κB as a therapeutic target in chronic inflammation: Recent advances. Mol. Med. Today.

[78] Hwang B.-M., Noh E.-M., Kim J.-S., Kim J.-M., Hwang J.-K., Kim H.-K., Kang J.-S., Kim D.-S., Chae H.-J., You Y.-O. (2013). Decursin inhibits UVB-induced MMP expression in human dermal fibroblasts via regulation of nuclear factor-κB. Int. J. Mol. Med..

[79] Yang G., Im H.-J., Wang J.H.-C. (2005). Repetitive mechanical stretching modulates IL-1β induced COX-2, MMP-1 expression, and PGE2 production in human patellar tendon fibroblasts. Gene.

[80] Gendrisch F., Esser P.R., Schempp C.M., Wölfle U. (2021). Luteolin as a modulator of skin aging and inflammation. Biofactors.

