# Supplementary material for: Oddioside A, a New Phenolic Glycoside Isolated from the Fruits of Morus alba (Mulberry), Protects TNF-α-Induced Human Dermal Fibroblast Damage
Source: Antioxidants (Basel). 2022 Sep 24;11(10):1894. doi: 10.3390/antiox11101894 (PMC9655652; doi:10.3390/antiox11101894)

Supplementary Materials

Contents

General Experimental Procedures

Acidic Hydrolysis of **1** and Sugar Identification

Figure S1. HR-DART-MS spectrum of compound **1**

Figure S2. ^{1}H-NMR spectrum of compound **1** (500 MHz, methanol-d_4).

Figure S3. ^{13}C-NMR spectrum of compound **1** (125 MHz, methanol-d_4).

Figure S4. ^{1}H-^{13}C HSQC spectrum of compound **1**.

Figure S5. ^{1}H-^{1}H COSY spectrum of compound **1**.

Figure S6. ^{1}H-^{13}C HMBC spectrum of compound **1**.

General Experimental Procedures

NMR spectra were obtained using a 500 MHz spectrometer (JEOL, Tokyo, Japan) and chemical shifts are expressed as δ values. Melting points were measured on an MPA 100 instrument (Stanford research systems, Sunnyvale, CA, USA) in open capillary tubes. UV spectra were obtained on an Optizen pop (Mecasys, Daejeon, Korea) apparatus. Optical rotations were evaluated on a Jasco P-2000 polarimeter (JASCO, Tokyo, Japan) using a 10-cm microcell. The high-resolution direct analysis in real time mass (HR-DART-MS) measured by an AccuTOF® single-reflectron time-of-flight mass spectrometer (JEOL Ltd., Tokyo, Japan) equipped with a DART ion source (IonSense, Saugus, MA, USA), and was operated with Mass center version 1.3.7 software. TLC analysis were performed on silica gel 60 F254 (Merck, Kenilworth, NJ, USA) and RP-18 F254S (Merck) plates. Compounds were visualized by dipping plates into 20% (v/v) H_2SO_4 reagent (Aldrich, St. Lois, MO, USA) and then heated at 110℃ for 5-10 min. Diaion HP-20 (Mitsubishi, Tokyo, Japan), Sephadex LH-20 (Amersham Pharmacia Biotech, Amersham, UK) and Silica gel (Merck, 60A, 70-230 and 230-400 mesh ASTM) were used for column chromatography. Flash chromatography was performed using a flash purification system (Combi Flash Rf; Teledyne Isco, Superior St, Lincoln, USA). Pre-packed cartridges (Redi Sep-C18, 13 g, 26 g, and 43 g; Teledyne Isco) were used for flash chromatography. All solvents used for the chromatographic separations were distilled before use.

Human dermal fibroblasts (HDFs) were obtained from PromoCell GmbH (Sickingenstr, Heidelberg, Germany) and used in the experiment. For cell culture, Dulbecco's modified Eagle medium (DMEM; Corning, Manassas, VA, USA) supplemented with 10% fetal bovine serum (FBS; Atlas, Fort Collins, CO, USA), 1% pen strep (penicillin/streptomycin; Gibco, Grand Island, NY, USA) was used and HDFs were cultured in a humidified incubator maintained at 37℃ with 5% CO_2.

Acidic Hydrolysis of 1 and Sugar Identification

Compound **1** (1.0 mg) was subjected to an acid hydrolysis with 1N HCl at 80℃ for 3 h. Sodium bicarbonate was added to stop the reaction. The reaction mixture was evaporated in vacuo. The absolute configuration of glucose and rhamnose in **1** was confirmed by the method from Tanaka et al [18]. Hydrolysate was dissolved in pyridine (500 μL) and L–cysteine methyl ester hydrochloride (1.2 mg) was added and heated at 80℃ for 1 h. The mixture of **1** was heated again at 80℃ for 1 h after adding σ-tolyl isothiocyanate (100 μL) and analyzed directly by HPLC under gradient system [A: 0.1% (v/v) formic acid in water, B: 0.1% (v/v) formic acid in acetonitrile, 10 to 50% B, 45 min]. The reaction mixture of 1 was detected at 31.8 and 35.6 min. The retention times of authentic D-glucose and L-rhamnose were 31.6 and 35.6 min, respectively, under the same conditions. Therefore, the absolute configuration of β-glucose in **1** was confirmed as D. Additionally, absolute configuration of the rhamnose was confirmed as L.

Figure S1. HR-DART-MS spectrum of compound **1**.

MOAL-K28_[M+Na]+

Data:210108_RH_PV1000_DV1950
Sample Name:
Description:
Ionization Mode:ESI+
History:Determine m/z[Peak Detect[Centroid,5,Area];Correct Base[3.0%]];Correct Base[5.0%];Average(MS[1] 1.71...

Acquired:1/8/2021 1:52:40 PM
Operator:Administrator
Mass Calibration data:210108_1
Created:1/8/2021 1:58:44 PM
Created by:Administrator

Charge number:1 Tolerance:5.00(mmu) Unsaturation Number:-1.5 .. 20.0 (Fraction:Both)
Element:^{12}C:0 .. 100, ^{1}H:0 .. 200, ^{23}Na:1 .. 1, ^{16}O:13 .. 13

Mass	Intensity	Calc. Mass	Mass Difference (mmu)	Possible Formula	Unsaturation Number
499.14202	481764.38	499.14276	-0.74	$^{12}C_{20}{}^{1}H_{28}{}^{23}Na_{1}{}^{16}O_{13}$	6.5

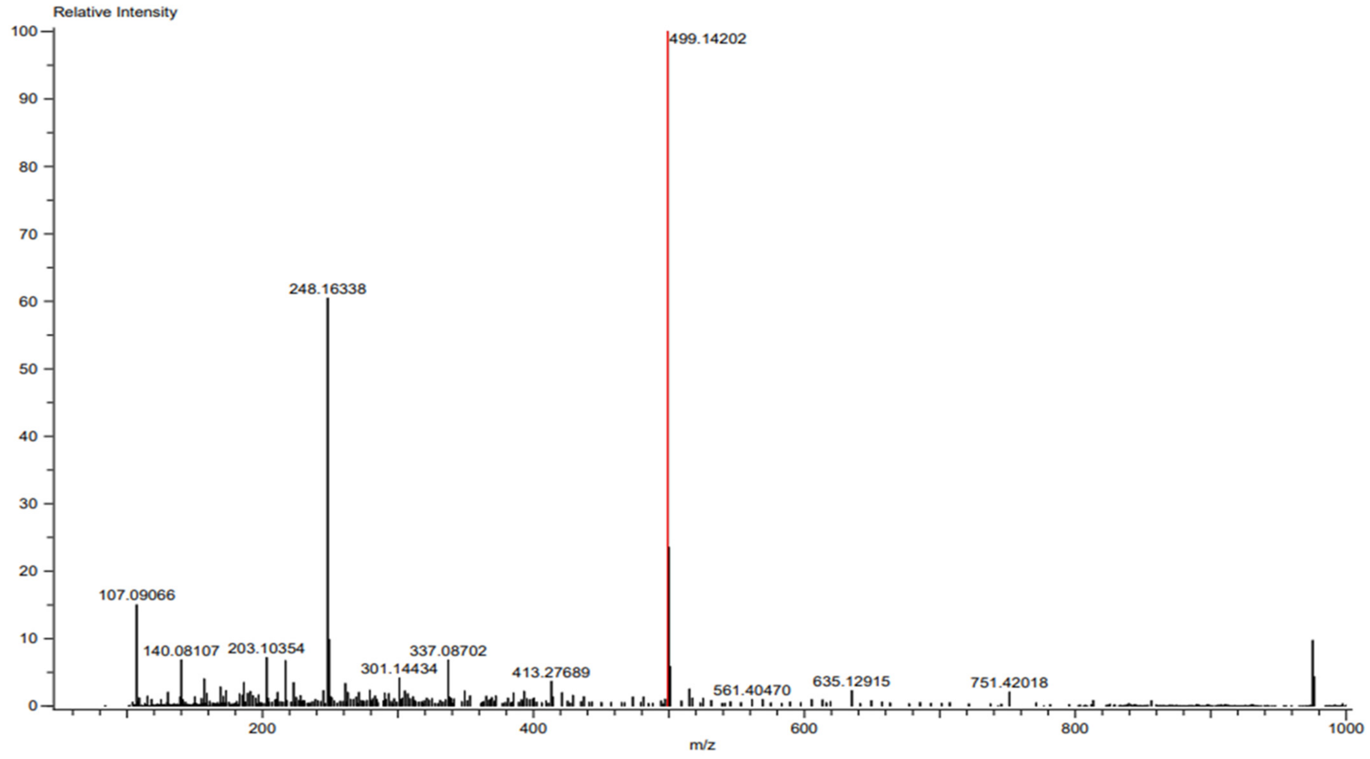

Figure S2. ^{1}H-NMR spectrum of compound **1** (500 MHz, methanol-d_4).

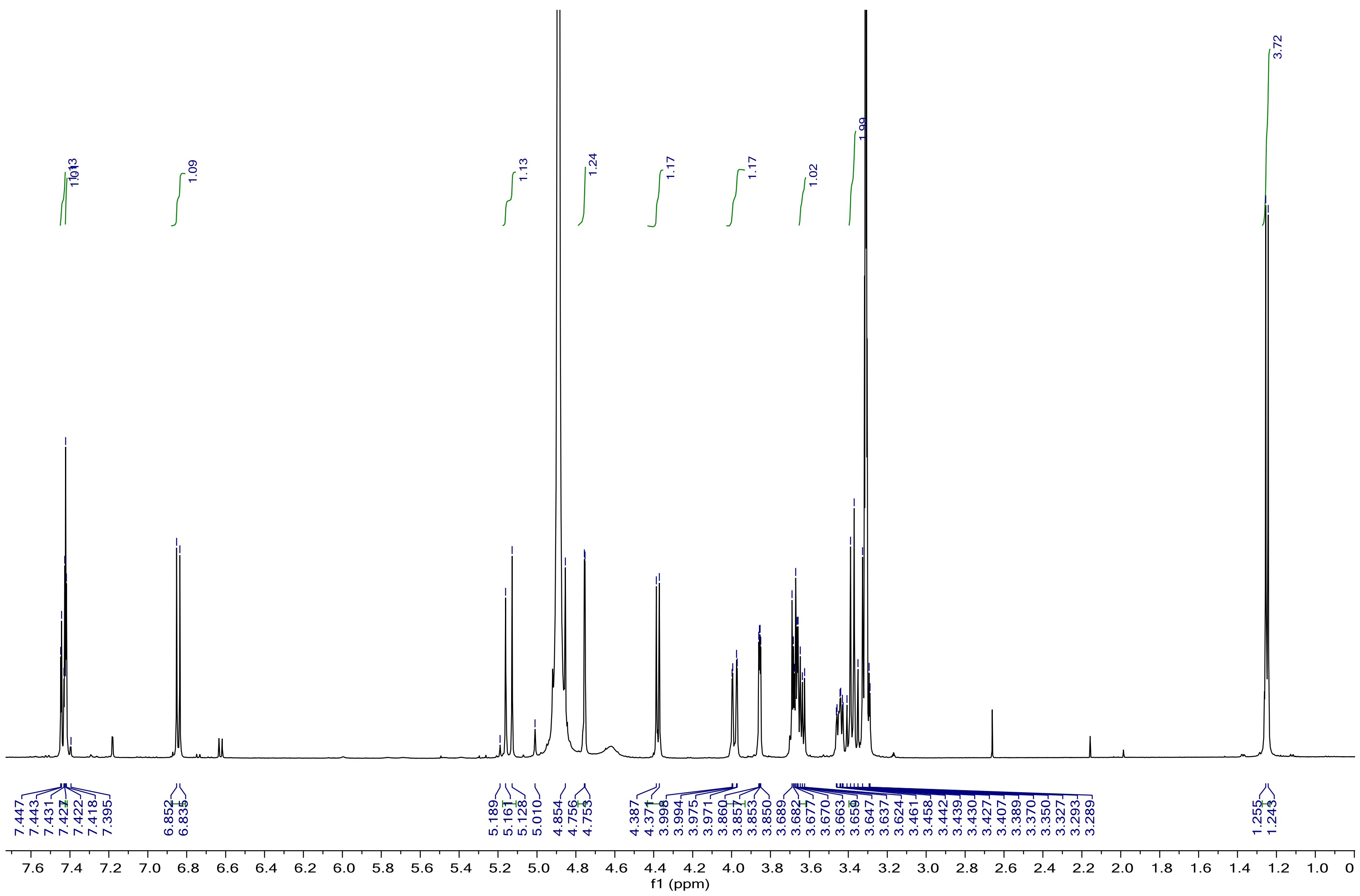

Figure S3. ^{13}C-NMR spectrum of compound **1** (125MHz, methanol-d_4).

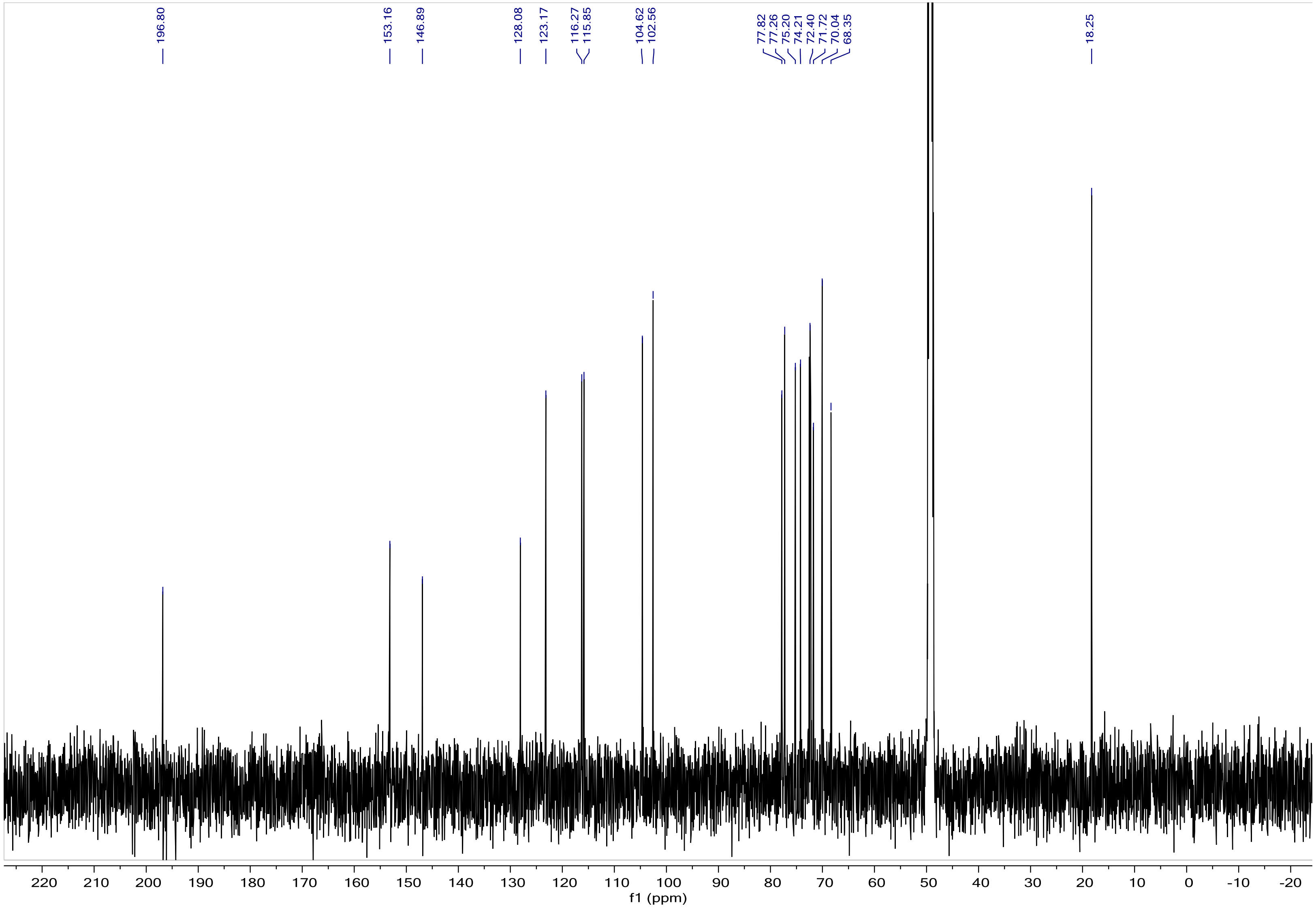

Figure S4. ^{1}H-^{13}C HSQC spectrum of compound **1**.

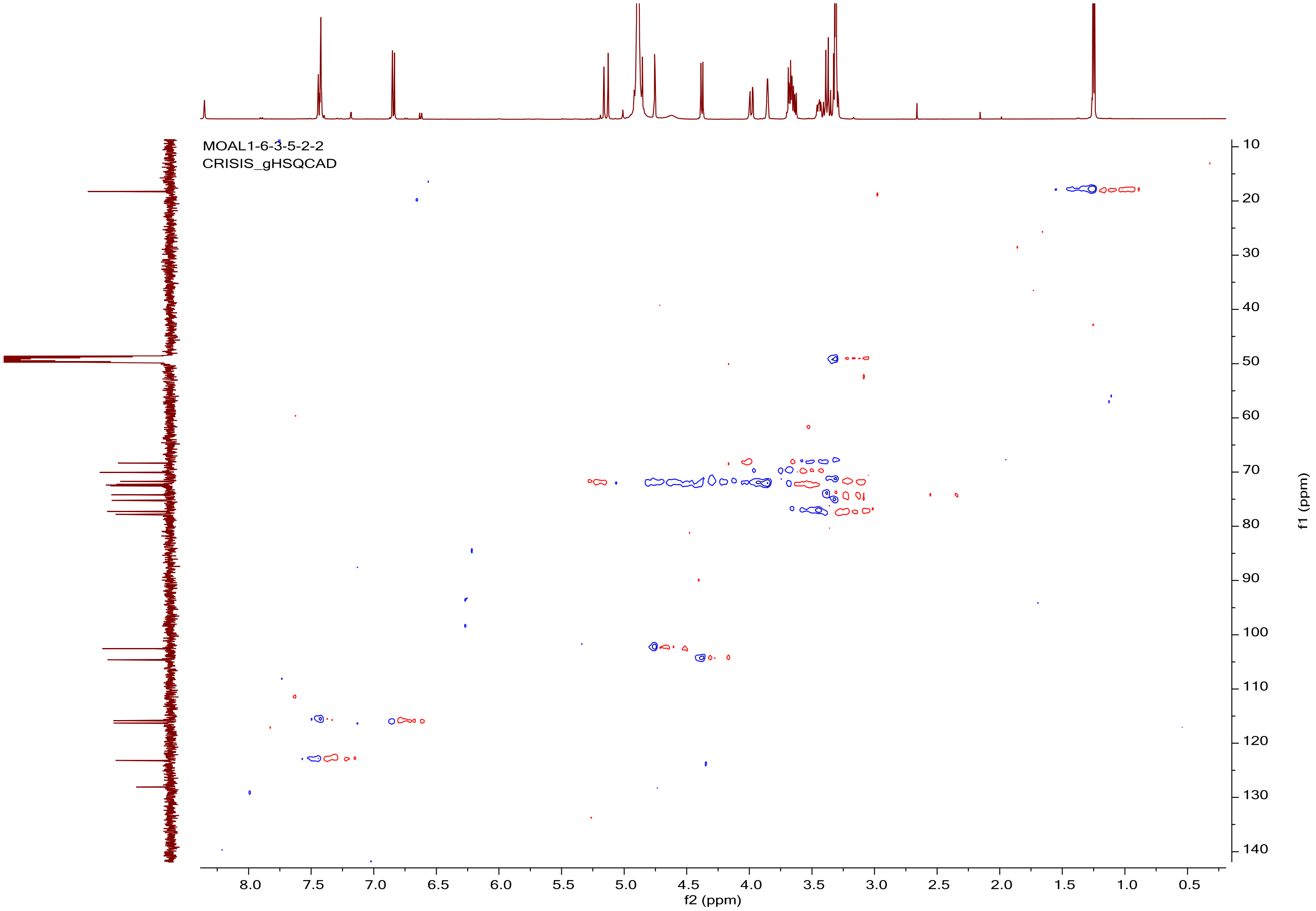

Figure S5. ^{1}H-^{1}H COSY spectrum of compound **1**.

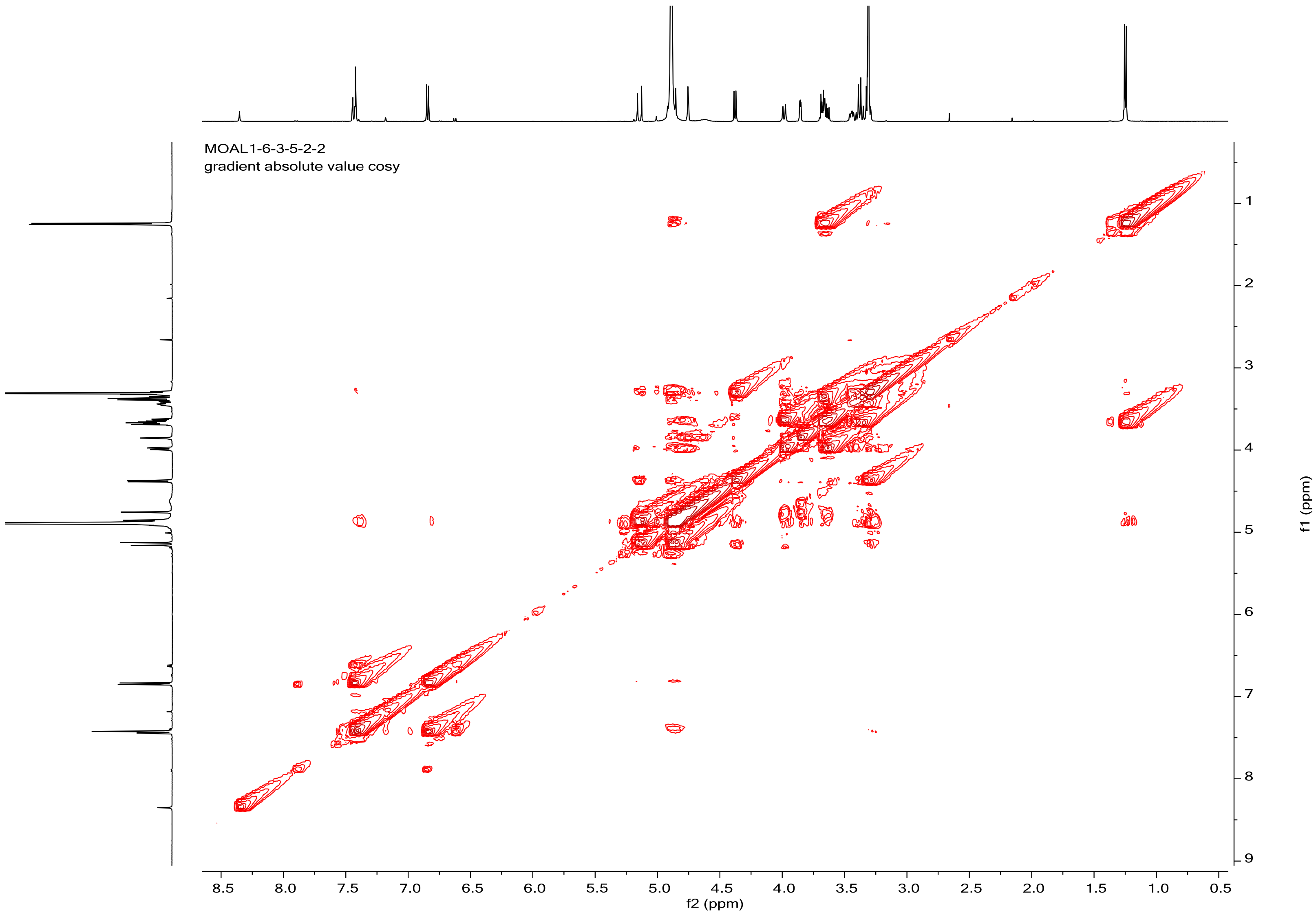

Figure S6. ^{1}H-^{13}C HMBC spectrum of compound **1**.

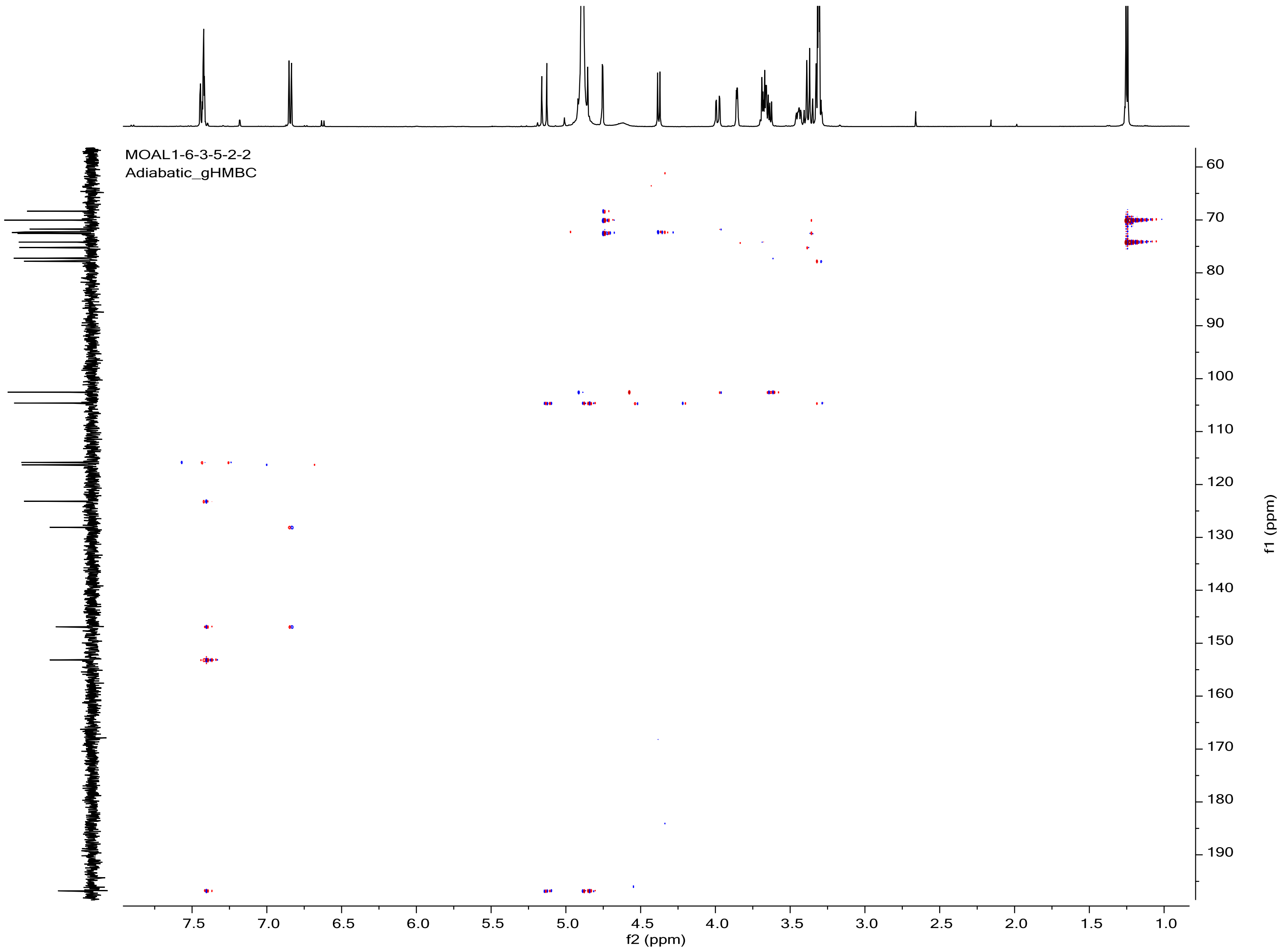

Supplement: Supplementary file 1 [file antioxidants-11-01894-s001.zip › antioxidants-1887963-supplementary.pdf]
